# *“Is she pregnant with Jesus?”* exploring sociocultural obstacles to following medical advice in the context of stillbirth prevention in Nigeria

**DOI:** 10.1186/s12884-025-07646-5

**Published:** 2025-05-20

**Authors:** Uchenna Gwacham-Anisiobi, Adetola Oladimeji, Victoria Yesufu, Jennifer J. Kurinczuk, Manisha Nair, Jenny McLeish

**Affiliations:** 1 National Perinatal Epidemiology Unit (NPEU), Nuffield Department of Population Health, University of Oxford, Old Road Campus, Headington, Oxford, OX3 7LF United Kingdom; 2 Institute of Health Systems Solutions and Virtual Care, Women’s College Hospital, Toronto, Canada; 3 Department of Geography and Environmental Management, University of West England, Bristol, United Kingdom

**Keywords:** Pregnancy, Antenatal care, Childbirth, Community influence, Health behaviour

## Abstract

**Background:**

Each year 182,000 babies are stillborn in Nigeria, representing nearly 10% of the annual global stillbirth burden. Imo state in south-eastern Nigeria has one of the highest levels of maternal health service access in Nigeria, yet this has not translated into good pregnancy outcomes. Many stillbirth prevention initiatives in Nigeria focus on maternal health education but empirical evidence suggests that sociocultural factors impact healthcare choices and outcomes. This study aims to explore women’s and health workers’ perspectives of the sociocultural barriers to following medical advice during pregnancy and childbirth, and specifically how these barriers may contribute to an increased risk of stillbirth. This study is part of a broader community-based stillbirth prevention mixed-methods research in Imo State, Nigeria.

**Methods:**

A qualitative descriptive study was conducted using in-depth interviews and focus group discussions. 38 participants were purposively recruited; 20 women and 18 health workers. Audio recordings were transcribed, translated and analysed using inductive thematic analysis.

**Results:**

Four themes were identified: (1) trust, where scepticism about health worker motives or competence and trust in community informal networks were highlighted (2) power dynamics within families, with husbands and older female relatives influencing health decisions; (3) personal and community beliefs that undermine confidence in medical interventions, including a pervasive stigma associated with caesarean section; and (4) grassroots proposals for solutions, emphasising the importance of a whole-community approach to maternal health education, mobilising peer voices, engaging traditional leaders and training of traditional birth attendants.

**Conclusion:**

This study provides insights into the sociocultural barriers to following medical advice during pregnancy in Nigeria, which include a lack of trust in health professionals, power dynamics within a woman’s family, and entrenched cultural and religious beliefs that oppose medical intervention. Women’s decisions about pregnancy and childbirth are heavily influenced by family and cultural norms. Culturally sensitive, community-wide interventions which aim to rebuild trust in the health system, involve women as decision-makers in antenatal care, and engage religious and traditional leaders would be beneficial for improving outcomes.

**Supplementary Information:**

The online version contains supplementary material available at 10.1186/s12884-025-07646-5.

## Background

A stillbirth, as defined by the International Classification of Diseases 11th Revision, is a baby born with no signs of life at 22 or more weeks of gestation, but for international comparison, the World Health Organization defines stillbirths as those occurring at 28 or more weeks, which is the definition adopted in this study [1]. About 182,000 babies were stillborn in Nigeria in 2021, representing nearly 10% of the annual global stillbirth burden [2, 3]. The stillbirth rate in Nigeria is 42 per 1,000 total births [4], markedly higher than the global average of 13.9 per 1000 births [2]. Stillbirth prevention interventions have been implemented in Nigeria [5–8], but progress in preventing stillbirths remains slow, with an annual reduction rate of only 2.3% between 2000 and 2019 [2]. The slow progress in stillbirth reduction in high-burden countries, including Nigeria, has been attributed to several factors, including lack of access to skilled and high-quality care during pregnancy and childbirth [9, 10]. In particular, the risk of stillbirth is increased when women receive fewer than four antenatal appointments, when facilities are poorly equipped, when there are critical shortages of skilled health professionals, when there is limited access to emergency obstetric care and when obstetric complications such as pre-eclampsia, prolonged labor, and fetal growth restriction are not adequately managed [11–13]. Access to obstetric care may be affected by multiple factors including the availability and affordability of care and transport to reach services [14, 15].

Imo State in south-eastern Nigeria has one of the highest levels of maternal health service access in the country [9, 16]. In 2018, 97% of pregnant women had four or more antenatal visits, 94% had skilled birth attendance, and 95% had a health-facility childbirth [9]. However, this high rate of access has not translated to the expected lower rate of maternal and perinatal complications and deaths. Two tertiary facility studies conducted in Imo State in 2012 reported stillbirth rates of 60–180 per 1,000 births [17, 18], while a 2022 study in 20 secondary hospitals found a rate of 38 per 1,000 births [19]. This persistent paradox of high access rates but poor pregnancy outcomes suggests that factors beyond healthcare access play crucial roles in maternity outcomes, as suggested by a nationwide survey in 2013 [20]. There is therefore need to explore additional factors in settings like Imo state with seemingly high levels of access to care. These factors might be institutional (related to the quality of care offered in health facilities) [21] or community-based (factors leading women not to act on medical advice regarding health during pregnancy and safety during childbirth) [22, 23]. Similar patterns have been reported in other low-resource settings across Africa and Asia [24–26].

Globally, many stillbirth prevention initiatives focus on maternal health education to promote positive health behaviour [5–8]. However, empirical evidence shows that sociocultural factors such as a lack of autonomy in decision-making, religion, and socio-economic status can impact healthcare choices, and therefore having access to health information may not automatically change women’s apparent pregnancy choices and thus outcomes [18]. These issues for women in many low and middle income countries have also been found to affect women in Nigeria [19–21], but it is not known whether or how these issues may contribute to the high stillbirth rate in Imo State [27–30]. This study explores women’s and health workers’ perspectives of the sociocultural barriers to following medical advice during pregnancy and childbirth, and specifically how these barriers may contribute to an increased risk of stillbirth. Understanding the specific local barriers from both women’s and health workers’ perspectives is essential for developing targeted interventions to reduce stillbirths in Imo State. ‘Medical advice’ is used in this study to include advice about pregnancy health, about using the antenatal and intrapartum care services available from trained healthcare workers, and about critical medical interventions. This study is part of a broader mixed-methods research of community-based stillbirth prevention in Imo State, Nigeria [6, 19, 31, 32].

## Methods

This study used qualitative descriptive methodology, theoretically informed by a contextualist epistemology [33]. This design was chosen because the intention was to explore participants’ own perspectives arising from individual contexts rather than to apply or generate theory, while also acknowledging the role of researchers’ interpretations in the production of knowledge and the researchers’ responsibility to ground findings closely in participants’ experiences [34, 35]. The research team consists of people with medical and health services research backgrounds, including Nigerians and non-Nigerians, and we recognise our positionality in shaping the research process. We maintained reflexivity throughout the study through regular team discussions to mitigate potential biases from our backgrounds, experiences or disciplinary training, and maintained an audit trail [36].

### Study setting

The setting was Imo State, southeast Nigeria, with an estimated population of 5,459,300 in 2022 [37]. The inhabitants are mostly Christians (90%) from the Igbo ethnic group [38, 39]. According to a 2018 survey, 91.4% of women of reproductive age have received secondary education or higher [9]. Imo State has a mixed healthcare system, with maternity care provided by government, private providers or religious missions, but only 1.7% of households had health insurance coverage in 2019 and healthcare expenditure exceeds 10% of household income for 68.6% of households [18]. Informal maternity care is available from unqualified traditional birth attendants [39]. Imo State’s healthcare organisation closely resembles the federal government’s three-tier system. The state has 563 primary health care facilities, 602 secondary health care facilities and two tertiary facilities [39] Over 70% of women in the state use private maternity services [9]. As of 2018, the state had 36 doctors and 115 nurses or midwives per 100,000 population [39] which are significantly below the standard recommended health care worker to population ratio.

### Sampling and recruitment of participants

Purposive sampling [40] was used to select participants, who were women who had experienced (i) a stillbirth or (ii) a ‘near-miss’ severe fetal complication, and health workers providing maternity care (iii) in hospitals or (iv) in the community at primary health care centres.

The inclusion and exclusion criteria are shown in Table 1. All potential participants were screened for eligibility by UGA, using the listed criteria. Women’s eligibility was confirmed either through their medical records or by the health worker who had attended to them on hospital admission. Health worker eligibility was confirmed while discussing the purpose of the study with them.

**Table 1:** Studyinclusion and exclusion criteria

Inclusion criteria	o Women	o Women who had a stillbirth in secondary health facilities in Imo State between January 2020 to June 2023,
		o Women who had a ‘near-miss’ in secondary health facilities in Imo State between January 2020 to June 2023, as confirmed by a health worker. Near-miss was defined as women who had severe foetal complications that did not end in a stillbirth.
	o Health workers	o Doctors, midwives, and nurses involved in maternal and newborn care, based in secondary health facilities.
		o Community-based health workers involved in maternal and newborn care, based in primary health centres: nurses, midwives, community health extension workers.
Exclusion criteria	oWomen	o Mothers below 18 years old
		o Potential participants who did not consent to participate in the study
		o Women who had gave birth outside a secondary facility (including home births, births with traditional birth attendants and births in primary health centres) to ensure consistency in data and expected quality of care.
		o Volunteer participants whose account of “difficult childbirth” could not be confirmed by a health worker as meeting the study inclusion criteria.
	o Health workers	o Health workers who are not directly involved in providing maternal and newborn healthcare services.

To ensure that participants were drawn from a range of hospital types and locations, recruitment began at the twenty hospitals that were the setting for the wider research project. A detailed description and justification of the recruitment process for the twenty hospitals involved in the broader research project have been reported elsewhere [32]. Notices were also put out through the local government health department office, which is the administrative centre for community health workers stationed in primary health centres. However, due to the increased activities of separatist Biafra groups in Imo State at the point of data collection, participant recruitment was limited to local government areas considered ‘safe’ by local security intelligence agencies. Trained nurses at each hospital invited eligible women to participate and shared the contact details of those who agreed with the research team. Due to the sensitivity of the topic, many eligible women were unwilling to participate in this study, so a snowball approach [41] was also used, with women passing on the invitation to other women who had stillbirths at different facilities. Health workers were invited to participate through personal invitations (by email or telephone) from the interviewer and through notices about the study put up on hospital notice boards, aiming to reach both health workers who were already collaborators on the wider research project and others with no prior connection. All potential participants were given a copy of the participant information sheet and it was discussed with them by telephone or in-person at least 48 h before their interview or focus group. Informed consent was obtained from all participants, and all relevant guidelines and regulations were followed in conducting the study.

### Data collection

Participants took part in individual or paired semi-structured interviews or focus groups, between 5th May 2023 and 27th June 2023. Topic guides were developed based on the literature and with the support of a woman who had experienced stillbirth (see supplementary file). Pilot interviews were conducted with one health worker and one woman who had a near-miss, but no changes to the topic guides were necessary. The interview dates and modes (telephone/videoconference/face to face) were scheduled to suit each person. The focus group participants were grouped based on their preferences for date and mode of interview. Women and hospital-based health workers were interviewed individually due to the sensitivity of the topic and scheduling challenges. Most women opted to be interviewed in person in their homes or virtually, through a video or telephone call. All hospital-based health workers were interviewed in person in their offices or virtually. Community health workers took part in small focus groups or, where this was not possible, in paired interviews. One focus group (three participants) and one paired interview (two participants) were conducted after working hours at primary health centres chosen by participants, while one focus group discussion (three participants) was conducted virtually. Data saturation was attained for health workers– that is, there were no new insights or codes identified in the last interviews, indicating that additional data collection would not contribute further to the analysis. Data collection had to be stopped before data saturation was attained for women due to limited resources available for this DPhil project.

Participants were offered the choice of using English language, ’pidgin’ English, or Igbo language. All interviews were carried out by UGA, who is a medical doctor from an Igbo speaking state. She conducted the interviews with an awareness of how shared cultural knowledge can influence interviews and the importance of overcoming potential perceptions of power and status differentials.

The interviews and focus group discussions were audio-recorded and transcribed verbatim soon after completion of the interviews by two members of the research team fluent in all three languages. The transcripts were translated and back-translated from Igbo and pidgin English to English by UGA who is fluent in all three languages, to ensure cross-cultural equivalence and to preserve the participants’ meaning [42]. Details that could identify a person or a health facility were removed and participants were given pseudonyms beginning with SB (woman who has experienced stillbirth), NM (woman who has experienced near-miss), CHW (community-health worker) or HHW (hospital-based health worker). For safeguarding purposes, the medical team and clinical psychologists (where available) at the local hospital were sensitised about this study prior to the start of data collection. Participants were assured of their right to leave the interview at any point or decline to respond to any question, and the interviewer remained attentive to the potential for distress and prepared to refer those needing support.

### Data analysis

Inductive thematic analysis was carried out in parallel with ongoing data collection [43]. Transcripts were read and re-read for familiarity by UGA, AO, VY and JMc and were independently coded descriptively, using annotations in Microsoft Word. The codes were refined, combined and compared iteratively as data collection and analysis continued [44]. The technique of constant comparison was used to examine the differences and similarities between the perspectives of women and health workers, and within and between types of health worker and health facility [45]. Codes were developed into themes through discussion within the research team. The researchers remained reflexive throughout the data collection and analysis, mindful of their own cultural backgrounds, personal and professional perspectives and experiences of health and health-seeking behaviours during pregnancy and childbirth, and potential issues arising from UGA’s previous professional relationships with three of the health worker participants.

### Ethical statement

Ethical approval for data collection was obtained from the Oxford Tropical Research Ethics Committee (OxTREC Ref: 535 − 22) and Federal Medical Centre Owerri Ethical Committee (FMC/OW/HREC/VOL.II/68). Informed consent was obtained from all participants, and all relevant guidelines and regulations were followed in conducting the study.

## Results

### Participants

There were 38 participants: 10 women who had stillbirths (including two women who had two consecutive stillbirths), 10 women who had near-misses, 10 hospital-based health workers and eight community-based health workers. Interviews lasted 24–72 min (mean 39.9); 12 were in-person; and 18 were conducted virtually (by telephone or Zoom call). Focus group discussions lasted 46–61 min (mean 53.4). Twenty interviews were conducted in English, while four and six were conducted in pidgin and Igbo respectively. Three focus group discussions were conducted in English.

Participants’ socio-demographic characteristics are shown in Tables 2 and 3.

In the analysis, there were no substantial differences in the perspectives of participants from these four groups, so the findings are presented collectively to capture their shared experiences and insights.

**Table 2:** Sociodemographic characteristics of women included in the study

Pseudonym	Age range	Marital status	Parity	Childbirth affected	Level of education	Employment	Type of facility	Geographical location
SB01	36–40	Married	3	2nd	Tertiary	Self-employed	Public	Urban
SB02	31–35	Married	2	1st	Secondary	Self-employed	Private	Urban
SB03	25–30	Married	4	3rd and 4th	Secondary	Self-employed	Public	Rural
SB04	21–25	Cohabiting	1	1st	Secondary	Unemployed	Private	Rural
SB05	21–25	Married	1	1st	Tertiary	Civil servant	Private	Rural
SB06	21–25	Married	2	1st	Secondary	Self-employed	Mission	Rural
SB07	36–40	Married	4	1st	Tertiary	Teacher	Private	Urban
SB08	26–30	Married	3	1st (1 twin), 2nd	Secondary	Unemployed	Mission	Rural
SB09	21–25	Married	3	1st	Secondary	Hairdresser	Private	Rural
SB10	31–35	Married	2	2nd	Secondary	Self-employed	Private	Rural
NM01	21–25	Married	1	1st	Tertiary	Front desk clerk	Mission	Rural
NM02	31–35	Married	6	6th	Secondary	Technician	Mission	Rural
NM03	31–35	Married	3	3rd	Tertiary	Nurse	Private	Urban
NM04	21–25	Married	1	1st	Tertiary	Civil servant	Mission	Rural
NM05	31–35	Married	2	3rd	Tertiary	Nurse	Private	Rural
NM06	31–35	Married	4	2nd	Tertiary	Self-employed	Mission	Urban
NM07	21–25	Married	3	3rd	Secondary	Unemployed	Private	Rural
NM08	21–25	Married	1	1st	Secondary	Self-employed	Private	Rural
NM09	31–35	Married	2	2nd	Secondary	Self-employed	Public	Rural
NM10	36–40	Married	1	1st	Secondary	Self-employed	Public	Rural

**Table 3:** Sociodemographic characteristics of health workers included in the study

Pseudonym	Professional group	Years in service	Facility type	Geographical location
HHW01	Nurse/midwife	8	Mission Hospital	Rural
HHW02	Doctor	9	Public Hospital	Urban
HHW03	Senior Auxiliary Nurse	4	Mission Hospital	Rural
HHW04	Doctor	21	Private Hospital	Rural
HHW05	Nurse/midwife	14	Mission Hospital	Urban
HHW06	Nurse/midwife	15	Public Hospital	Rural
HHW07	Doctor	5	Mission Hospital	Rural
HHW08	Nurse/midwife	7	Private Hospital	Urban
HHW09	Nurse/midwife	14	Private Hospital	Rural
HHW10	Doctor	9	Mission Hospital	Rural
CHW01	Nurse/midwife	16	Primary Health Centre	Rural
CHW02	Nurse	5	Primary Health Centre	Rural
CHW03	Community health extension worker	8	Primary Health Centre	Rural
CHW04	Health Assistant	4	Primary Health Centre	Rural
CHW05	Community health extension worker	3	Primary Health Centre	Rural
CHW06	Nurse	12	Primary Health Centre	Rural
CHW07	Food science officer	7	Primary Health Centre	Urban
CHW08	Nurse	6	Primary Health Centre	Rural

### Findings

Four themes were developed from the data. Three themes describe the reasons why women with access to formal maternity care might nonetheless not follow medical advice that could potentially reduce the risk of stillbirth. These three themes cover trust, power within families, and personal and community beliefs that undermine confidence in medical interventions. The fourth theme describes participants’ proposals for community-based solutions. A series of sub-themes were developed within each theme.

### Theme 1: trust

Women in Imo State have access to pregnancy advice and care from medical and non-medical sources. The subthemes *Lack of trust in healthcare workers* and *Trust in community expertise* reflect the essential role of trust in motivating women to act upon the information they received, which affected their choices for both antenatal and intrapartum care.

#### Lack of trust in healthcare workers

Participants described how some women were cynical about pregnancy advice given by healthcare workers, who they did not trust to be acting in the best interests of mother and baby:

*“You give them the medications and they throw them away thinking that maybe they are outsmarting the doctor. But they don’t know they are only short-changing themselves.”*(SB03).

In particular, a woman might refuse a caesarean section if she believed that the doctors were recommending this expensive operation for monetary gain:

*“They think that many doctors are sectioning people because of money. Before you say anything*,* they [the women] have started shouting.”* (CHW04).

Women participants said that some women in their communities also considered antenatal visits a “*waste of time*” (SB02) or a “*waste of money*” (NM01) if they felt well, because they were not aware that the purpose of antenatal visits included early identification of problems.

*“When you’ve had your first and second baby*,* you start telling yourself that you are already a mini doctor*,* what is there that I don’t know about?”* (NM06).

Distrust of health professionals could be compounded in two ways. Firstly, the women described many instances of verbal and physical abuse, abandonment and discrimination that they either personally experienced or witnessed during antenatal care and childbirth in hospitals. This directly undermined women’s trust in health professionals and their ability to access essential pregnancy information.

*“Speaking harshly to women will make them suppress their questions. They will be dying in silence.”* (NM04).

Experiencing disrespectful or neglectful maternity care at a health facility made some women feel so unsafe that they abandoned the facility for informal care.

*“Some of the health workers are very*,* very aggressive to our pregnant mothers*,* and it’s not good. When you are aggressive to them or the way you talk to them will even make them leave the facility*.”(CHW08).

*“There was the time I was really very sick in my early pregnancy. So I went to the health centre to look for the nurses; my sister and I were waiting for the nurses to come attend to us. Meanwhile*,* they were at the backyard watching a movie on their phone and we were seriously waiting for them. (…)It was really that sort of character that made me change from using the Primary Health centre to the maternity [traditional birth attendant].” (NM08)*.

Secondly, a hospital’s reputation within the family or community could also weaken a woman’s confidence in using the facility.

“*Someone came to my shop to tell me her experience of almost losing her baby… They were calling the nurse to come*,* that the baby was already coming*,* and the nurse was telling her that if the baby was coming out*,* she wouldn’t even have the sanity to be talking about it. Why will anyone want to go [to that hospital]?”* (NM06).

Healthcare workers observed that delayed intrapartum referral from a traditional birth attendant to the hospital was a factor in some stillbirths which were then recorded as occurring in the hospitals. When such a pregnancy ended with a poor outcome, the news tainted the reputation of the facility where the stillbirth or other adverse event occurred. This created a negative feedback loop in which communities attributed the poor outcome to a lack of skill on the part of the healthcare workers at the hospital, so women were even less likely to go directly to the hospital for birth, creating the risk of further late referrals ending in poor outcomes.

*“When they hear this particular bad story*,* it keeps ringing in their heads…The doctor is telling you*,* ‘Come to the hospital*,*’ but you wouldn’t go there because you are scared*,* because you heard that [name]’s mum died while putting to bed there*,* not even knowing the circumstances surrounding her death.”* (HHW10).

#### Trust in community’s expertise

Participants described the sense of collective responsibility towards pregnant women in their communities, and women said they constantly received unsolicited pregnancy advice from members of the community. Some felt social pressure to be seen to act on this advice, even when they were aware that this might be “*like the blind leading the blind.”* (NM01).

*“When it’s your first baby everybody will want to tell you what to do and what not to do. It’s very hard because the advice will keep coming even if you try to shun them*,* people will always keep telling you (…) you can’t run away from it.”* (NM06).

Most women said that they primarily relied on their mothers or other experienced relatives and friends for information. Although this was an important form of social support, participants said that women might make risky choices if they trusted inaccurate information from family or friends over evidence-based medical information.

*“Sometimes you learn from people that have given birth before you*,* even this method of learning usually misleads a lot of persons. Because most people will stick to the one they know even though it isn’t right*,* but because you are a first-timer you tend to believe them because you have not experienced it.*” (NM06).

Women were reported to particularly trust the recommendations of community members that were based on personal experience and local custom. For example, a woman might choose to have her maternity care from an untrained traditional birth attendant instead of at a hospital, based on their local word-of-mouth reputation for successful births.

*“So*,* the myth there is like they tell you*,* ‘Oh my mother*,* my grandmother and my great-grandmother was delivered in the house*,* and it was this woman*,* this particular old nurse that was taking all the deliveries and nothing [bad] ever happened*.’” (HHW10).

Reliance on traditional medicine and practitioners was potentially harmful for women and their babies. For example, in some communities it was considered normal to take ‘herbal concoctions’ to prevent umbilical cord coiling or reduce the weight of the babies, but health workers reported that these could cause fatal complications for the baby. Likewise, traditional birth attendants were believed by some communities to have exceptional skills, but health workers reported that their antepartum interventions, such as an attempt at turning a breech baby, could inadvertently lead to stillbirth.

*“Almost all of them visit the traditional birth attendant or the herbalist. Some may tell you they are going to take medicine that will untie the cord*,* some medicine that will make the baby to turn. This is somebody who has breech in late trimester. The person will be going to a native doctor or traditional birth attendant to turn the baby. And in the process of trying to turn the baby*,* they cause either APH [antepartum haemorrhage] or getting the cord to tangle.*” (HHW04).

This trust could extend to taking advice from social media groups focused on maternal health, and misplaced trust could lead to stillbirth when community members recommended traditional remedies instead of medical help.

*“There was a case [of misinformation] that happened that she [pregnant woman] told them*,* her baby is not too fine on Facebook*,* and she was advised to go and grind uda [negro pepper] and uziza seed [West African black pepper] and pour it inside schnapps [alcoholic drink] and drink*,* that the baby will be bouncing.”* (*CHW07*).

### Theme 2: power in the family

Many pregnant women in Imo State live in families where the individual does not have the agency to make her own health choices. The communal family structure and power dynamics within a woman’s home may affect her health and her pregnancy both directly (reflected in subtheme *Family attitudes to pregnancy health*) and indirectly by controlling her access to healthcare (reflected in subtheme *Families as maternity decision makers*.)

#### Family attitudes to pregnancy health

Health workers highlighted that some families did not prioritise women’s health in pregnancy, and made no efforts to minimise stress and maximise nutrition. In a setting where a woman was expected to do hard chores while pregnant, it might be difficult for the woman to advocate for her own needs and follow medical advice.

*“In the morning*,* they [woman and her husband] will eat garri [granulated cassava]. After garri*,* they will go to the farm. When they come back*,* even without taking a bath*,* they will ask the woman to go and cook. With that stress in pregnancy*,* no good meal*,* financial problems in the family… it can lead to [stillbirth].”* (CHW01).

Relatives who had been pregnant themselves were reported to maintain this cultural attitude by specifically objecting to any preferential treatment for another pregnant woman.

*“But when the family*,* if all they know is to pass comments like ‘She should go and cut up firewood*,* is she pregnant with Jesus? When I was pregnant*,* I was climbing things*,* I was climbing heights*,* so she should do chores.’”* (HHW09).

#### Families as maternity decision makers

In families where health decisions are not considered to be an individual matter, pregnant women are expected to defer to their husband, mother or mother-in-law. If another family member is the controlling decision maker, they may enforce traditional practices against a woman’s will, such as avoiding any antenatal check-ups in the first half of pregnancy.

*“Then she [pregnant woman] will be dying in silence o*,* because she is under the control of the mother-in-law or the relatives of the husband. So they won’t allow her to go to the hospital. They will say it’s too early. They will wait until six months or seven months…In the process*,* no routine drug o*,* no good diet o*,* no exercise o*,* nothing! No health education*,* so all those things affect them.”* (CHW01).

Participants described how the older generation could deny pregnant women access to formal antenatal care altogether. Even if they were allowed to register for antenatal care at a hospital, at the time of birth women were sometimes pressured into using facilities preferred by their mother or mother-in-law.

*“She wanted to register in a hospital*,* but her mother-in-law emphatically told her that she should register in a maternity [traditional birth attendant] as her son [the husband] does not have that kind of money to register her in a hospital.”* (NM02).

*“The last stillbirth we had*,* her mother took her to a native doctor*,* where the woman stayed for 2 days. (…) She is registered in [the hospital] and has been coming for antenatal*.” (HHW01).

When facing an emergency, health workers reported that some women need the permission of their husbands in order to consent to treatment.

*“Many women*,* by the time you try to explain to them*,* they will tell you*,* ‘Doctor*,* wait. I have to relay to my husband first; let him come and decide. Whatever my husband says*,* that is it.’”* (HHW02).

Their husbands may also need the permission of other family members or may need to consult with their religious leaders to give their blessing before they consent to treatment, all of which can delay a time-critical intervention.

*“Even when the husband comes*,* sometimes the husband will still have to wait for another relative to come before they will now take decision. Sometimes*,* they even wait for their religious or spiritual leaders to help them make decisions*.” (HHW07).

Where doctors recommended an expensive intervention such as emergency caesarean section, couples who could not afford this might ask for financial support from a wealthier family member not present in the hospital. This could cause further delays in the decision-making, with the outcome dependent on the relative’s opinion of the intervention.

*“The woman does not want any bad outcome; the man doesn’t want to hear anything that goes wrong with his wife*,* but he is not the one financially stable to pay…now his neck and his hands are tied. He goes back to maybe a brother-in-law or mother-in-law who is*,* in quote*,* like ‘the decision maker’. And of course*,* that one will tell you*,* ‘All my children*,* I gave birth to them vaginally*,* what do you mean?’”* (HHW02).

### Theme 3: beliefs that undermine confidence in medical interventions

This theme explores three types of beliefs that could affect women’s or families’ willingness to consent to interventions which could potentially avoid stillbirth, reflected in three subthemes.

#### Straightforward birth as a sign of womanhood

Participants said that some communities prized straightforward birth as a sign of womanhood, and this cultural norm could be reinforced by social pressure and directly influence women to take decisions during labour that put their babies at risk. Healthcare workers said that some women wait at home until their labour has significantly progressed before they come to the facility, in order to be able to boast of a brief labour; if they develop complications at home, this increases their chance of having a stillbirth.

*“They use it to talk when they go back to the village. [Mimicking mothers] ‘Labour was not difficult for me; immediately I arrived*,* I gave birth. I didn’t even wait up to 2 minutes.’ They never knew that you have to be monitoring both mother and baby in labour.”* (CHW03).

All participants reported that there is a marked sociocultural stigma associated with caesarean sections in many communities. Fear of being publicly “*mocked*” (HHW01) for weakness could influence the actions taken by a woman and her family when a caesarean section was recommended.

*“Sometimes it’s because they feel people will castigate them. The village people will say*,* ‘She was unable to push out her own child. She is unable to bear her own child.’”* (CHW04).

#### Fears about caesarean section

Far from understanding caesarean section as an intervention that might save their baby’s life, it was feared by some mothers. Participants described how in some communities (and reinforced by local practice in some hospitals), spontaneous vaginal childbirth was called “*safe delivery*”, implying that other forms of birth are not safe. Some health workers opined that this belief may influence how quickly a woman consents to childbirth interventions.

*“They are not considering safe delivery as healthy mother*,* healthy baby*,* irrespective of the fact whether it is vagina or CS [caesarean section]. For them the definition of safe delivery is a woman that delivered vaginally*,* but once you deliver via CS*,* it is no longer a safe delivery.”* (HHW02).

Some mothers were reported to believe (incorrectly) that having a caesarean section would automatically mean that all subsequent births must be by caesarean. When a caesarean section was recommended, health workers said that some women therefore “*run away*” (HHW07) from the facility to a traditional birth attendant who has promised to help them achieve a vaginal birth.

*“Some women say*,* ‘They will hurriedly only offer CS and put fear into you*,* and it is now CS forever. That means all of your childbirth and delivery will now be CS. So*,* because of that*,* don’t you ever come near a hospital’*.” (HHW02).

*“After looking at the baby*,* you will be asked [by the doctors] to go for CS to save your baby and yourself. But those maternity staff [traditional birth attendants]*,* they will not advise CS*,* to prove that they are old nurses and have taken delivery of babies since before Jesus.”* (SB01).

Health workers said that some communities believed women should avoid medical facilities for their first childbirth on the grounds that health workers may pressurise them into having a caesarean section if labour did not progress quickly.

*“There is this notion that it is not good for a first-time pregnant mother to come to the hospital or orthodox centre [formal health facility] to deliver. They will tell you that because it’s your first time*,* they will not be patient with you to allow you to go through the duration of labour (…) they will hurriedly only offer CS.”* (HHW02).

#### Religious faith and objection to interventions

Religious beliefs and practices were reported to shape many women’s and families’ engagement with maternity care, including during medical emergencies that could lead to stillbirth. In some cases this meant turning to prayer before, or instead of, seeking a medical solution.

*“Once somebody is sick (…) the family will take the person to church first for prayers before they come to the hospital. So*,* the person might die there at church or die on their way to the hospital. So that time wasted means a lot.”* (HHW07).

Some health workers described how the risk of stillbirth was increased when trained health workers also shared cultural norms which made them slow to recognise danger signs or prone to suggesting traditional remedies. For example, some communities considered conditions such as eclamptic fits to be spiritual issues requiring spiritual interventions.

*“They will always tell you that it [the sickness] is more spiritual*,* that even though I am a health worker*,* that does not mean that I have thrown away my traditional practices.”* (HHW02).

In some cases, a woman’s religious belief might lead her to refuse emergency intervention when offered. Participants described the influence of Christian denominations who opposed caesarean section on the grounds that Christians should give birth naturally *“like Hebrew women*” (SB07). Health workers reported that it was much more difficult to get consent for interventions when a woman had a faith-based objection.

*“She was counselled on CS*,* but she said ’it wasn’t her portion’ and had to go to a maternity home. When they had tried labour for like two days in the maternity*,* she had to come back to the hospital; unfortunately*,* before anything could be done*,* the baby was gone already.”* (HHW08).

These beliefs could be directly reinforced by the intervention of religious leaders. A community health worker described her personal experience of having to stand her ground when her pastor counselled her to refuse a caesarean section.

*“I was at [the] hospital*,* I was booked for CS. I called my pastor and said*,* ‘Please*,* I just need you to pray for me; this is what is happening.’ He said I should leave there immediately; I told him that I’m not going to leave because that’s the only way I can give birth. I’m due and the consultant said it has to be an emergency CS. So*,* you can imagine*,* some will listen to their pastors and go*.” (CHW04).

### Theme 4: grassroots proposals for solutions

This theme describes the solutions that health workers and women proposed to address the problems identified and has four sub-themes.

#### Work with families, communities and leaders

Recognising that women are not necessarily individual decision makers about pregnancy and birth, participants suggested that a whole-community solution was needed. This could begin with including families in maternal health education.

*“Once you explain the condition to the patient and the relatives*,* I think they will love to do that to save their lives.”* (NM05).

They also suggested that whole communities should be targeted in grassroots campaigns through markets and churches, to promote the use of maternity care including necessary interventions, tackle myths and end unsafe traditional practices. In Imo State it is traditional for women to return to their hometowns annually for “August meetings”, where women discuss community development and address social issues. These meetings are also a platform for skill acquisition and education, and participants suggested that leveraging these August meetings to offer maternal health education could reach both currently pregnant women and other women in the communities including the relatives of young women.

*“So it’s good that we visit churches’ community August meetings*,* sensitise them so that from there all these pregnant women will know what they are supposed to do.”* (CHW08).

Due to the strong influence of religion on women and their families’ decision to accept caesarean sections, health workers suggested bringing together religious leaders and healthcare workers to discuss issues at the intersection of religion and medical interventions.

*“Call those in health and those in the church and have a seminar or training with them to avoid them deceiving these pregnant women. Because these pregnant women sometimes they solely believe in them [religious leaders].”* (HHW05).

Participants also said that entrenched community attitudes had to be changed from within the community itself, and therefore outreach with traditional leaders is required to engage them in the work of preventing stillbirths.

*“The village clan or the village head… can help us communicate to them in languages they understand in order for the work to be easier for the health workers*,* and so that that message or that information can be disseminated properly to our mothers.”* (CHW05).

#### Mobilise the peer voice

As women are heavily influenced by the personal experiences of other women, healthcare workers suggested explicitly harnessing peer counselling for stillbirth prevention. They felt that inviting women to share their experiences of stillbirth at an antenatal education group was a powerful and relatable way of passing information to other women, if they are trained to do so.

*“She is now in second pregnancy*,* coming for antenatal and educating other women after the formal antenatal lectures. She says*,* ‘Whatever the doctors tell you*,* please comply o*,* don’t have my kind of experience.’”* (HHW02).

A peer example could also be used to reassure a woman who refuses what the health worker believes to be a life-saving intervention for her baby.

*“In cases where a woman adamantly refuses CS*,* it’s no longer one health worker who should handle it…Show her other women who had the same experience or did the surgery*,* and they were all fine. Allow her to have maybe one-on-one interaction with the persons.”* (NM05).

Two women suggested that training women community champions with accurate pregnancy health information would be another key way of reaching pregnant relatives, friends and neighbours.

*“There are already some women who have this kind of passion I have. It’s just for you to source for them and then teach them. Let them be aware and then the message grows.”* (SB07).

#### Improve trust in health workers through improved relational care

Some women believed that the reason why bad experiences of care remained unchecked in many medical facilities was because there was no culture of respect for pregnancy women and no adequate system for reporting poor behaviour. They called for each hospital to have a feedback system to capture women’s experiences and to actively use the findings to improve the quality of services.

*“They should have rules to make sure health workers take care of the patients and not be rude or harsh to the patients. Just like in [redacted] mission hospital now*,* the nurses are always calm to the patients*,* not because they want to but because of the rules that are there. It would help to calm situations down.”* (NM06).

#### Train traditional birth attendants

Health workers stated that since the women continue to trust the traditional birth attendants, it would be helpful for the government to design periodic training in basic skills for them. This would ideally enable them to understand the limits of their abilities, so they can recognise and make timely referrals in the event of intrapartum emergencies in their homes.

*“Due to some TBAs’ [traditional birth attendants’] level of knowledge*,* they may not know anything like that [danger signs]. So*,* you have to educate them; see in case you see something like this*,* know that it is not in a range that you can manage… Refer the patient where there is a doctor for expert management.” (*HHW09).

## Discussion

This study illustrates the complex sociocultural factors that influence women’s interaction with medical advice during pregnancy and childbirth in Imo State, Nigeria. The findings broadly align with existing literature on sociocultural barriers to maternal care in Nigeria [30, 46, 47], but additionally illuminate these issues through the specific lens of stillbirth prevention. Participants also provided valuable grassroots ideas for community-based solutions.

Trust plays a critical role in pregnant women’s healthcare behaviours, with women often relying on trusted family and friends they perceive as competent to help them navigate through pregnancy. Østergaard argues that an increase in trust offsets the power imbalance in healthcare, which enables patients to feel safe [48], but the findings here highlight a pervasive distrust of healthcare workers among pregnant women, consistent with previous research in Nigeria [49, 50]. When women believed that healthcare professionals were not competent and reliable, they assumed that medical advice was given for financial gain rather than in the interests of mother and baby. Distrust was intensified by experiencing, witnessing or hearing reports of verbal and physical abuse, neglect or discrimination. This raised the risks for stillbirth as women suppressed their concerns during interactions with healthcare workers or opted to continue their care with unqualified traditional birth attendants. Trust is fundamental to fostering effective engagement and collaboration between patients and healthcare providers especially in settings like Nigeria, where other concerns such as inadequate resources, corruption and past negative experiences can undermine the experiences of care. At the broader health system level, trust issues are widely reported and multifaceted, deeply impacting both service users and healthcare workers in Nigeria [51]. A critical example is the lack of trust in the government’s ability to improve working conditions fuelling the migration of competent health workers from areas of greatest need, exacerbating strain on the health system [52]. Similarly, the prevalent service user mistrust regarding the availability and quality of healthcare services, coupled with health workers’ scepticism about the government’s commitment to pay for services used by enrolees significantly affects engagement with Nigeria’s National Health Insurance Scheme (NHIS) [53].

Disrespectful maternity care has been reported to be common in Nigeria, including Imo State [49, 50]. Despite the extensive research of experiences of care in Africa, there are relatively few interventions addressing the gaps identified, and interventions have largely focused on health provider training, especially during health facility childbirth [54]. Evidence from the Subsidy Reinvestment and Empowerment Programme in Nigeria showed that interventions such as respectful healthcare attitudes, increasing skilled birth attendance, and provision of free essential drugs improved women’s institutional and interpersonal trust in health workers and significantly improved maternal health service utilisation [55]. This study adds the finding that mistreatment during antenatal care also influences women’s interactions with the available healthcare during pregnancy and birth and illustrates women’s desire to play a part in improving maternity care through the establishment of functional feedback systems that would enable them to report poor behaviour by healthcare workers so that this could be addressed at individual hospitals.

Delayed intrapartum referrals from over-confident traditional birth attendants could result in stillbirths that occurred or were identified in hospital. This, in turn, created a negative feedback loop where communities blamed the hospital for the stillbirths and believed it was safer to avoid hospital birth, creating the risk of further late referrals ending in poor outcomes. While a preference for TBAs over hospital births has been reported in other low- and middle-income countries [56–58], further research is needed to determine whether this vicious cycle observed in Imo State is present in other contexts and to explore its broader implications. Health workers proposed periodic training for traditional birth attendants as women continue to trust them with their care. The benefits of training traditional birth attendants remains contested [59–61]. However, the worsening shortages of health workers in Nigeria require mobilising and effectively using available human resources to make up for the shortfall [62]. In Timor Leste, a traditional birth attendant training programme was integrated into the national healthcare system and assessment studies suggest that the intervention contributed to the reduction of poor maternal and newborn health outcomes in the country [63].

The communal nature of society in Imo State strongly influenced all the findings and proposed solutions. On the one hand, the collective sense of responsibility towards pregnant women can be an asset for improving maternal, newborn and child health: pregnant women can tap into a broad social support system throughout pregnancy, potentially contributing to better outcomes [64]. On the other hand, the communal approach to pregnancy exposes women to misinformation from well-intentioned but poorly informed community members that may put them and their babies at risk, and social pressure to act on the misinformation. It also limits their autonomy as decision makers; and it shapes their desire to be able to display to their neighbours that they conform to an ideal of natural motherhood.

The findings that women may be unable to act on medical advice because of their limited autonomy, the culture of respect for elders and the intra-family power dynamics involving their husbands and older or wealthier relatives, are consistent with previous studies in Africa [47, 65]. Participants called for family-centred interventions which include involving key family members in health education and education about maternity care. A scoping review of interventions which engaged family members in maternal and newborn health care in low-income settings found that this can improve health outcomes and promote adherence to medical advice [66]. Other studies suggest that engaging family members in antenatal care can improve perceived social support, birth preparedness assistance, encourage positive maternal behaviours and improve outcomes [67, 68].

Maternal and newborn outcomes are undermined by some sociocultural and religious beliefs held by women and their families, which can lead them to avoid medical interventions. In keeping with findings from previous studies in Africa [46, 69], women in Imo State viewed short, non-interventional childbirths as a mark of womanhood, and social pressure to conform to this societal ideal led some women to make risky childbirth decisions. The well-documented stigma associated with caesarean section [46], which is driven by the fear of being perceived as weak or facing social judgement, hinders some women and their families from accepting this potentially life-saving intervention. This fear was worsened by the misconception that future childbirths after a caesarean section was only possible through caesarean section. This stigma is exacerbated by prevailing religious beliefs, especially among some Pentecostal denominations [70], that undergoing a caesarean section represents a lack of faith. For such women, phrases such as “*I shall deliver like the Hebrew women*” illustrated their strong belief in natural childbirth and divine intervention. These beliefs motivated some women and families to delay consent, to rely on prayer, or to choose to birth with the support of traditional birth attendants who had a positive reputation within their communities for achieving vaginal birth. This health behaviour is not unique to Nigeria; it has been reported in many West African and a few East African studies, and has been linked to adverse maternal and foetal outcomes [46, 71–73]. Participants highlighted that some health workers shared these cultural and religious norms, potentially hindering timely recognition and management of complications.

Recognising that individual pregnancies are embedded in these complex social systems; participants recommended a comprehensive whole-community approach to health education about pregnancy and birth. This would involve culturally sensitive interventions which tackle myths and emphasise the importance of timely care and the reasons why interventions are offered. The interventions would be based on sustained grassroots mobilisation, sensitisation through media and traditional community meetings, and working with traditional leaders. The effectiveness of such interventions in low-resource settings is well documented and can significantly improve maternal outcomes by addressing both general and specific health concerns [74, 75]. Participants suggested that promoting collaboration between healthcare workers and religious leaders could enable the integration of religious and medical perspectives of maternity care. This strategy has proven effective in increasing uptake of formal maternity care in other parts of Nigeria [76, 77]. Given the confidence women placed on experiential knowledge rather than the views of healthcare workers with professional training, participants also suggested training local women as peer counsellors and incorporating them into antenatal care. This method aligns with existing evidence that peer counselling can positively influence health behaviours [78].

These findings have important policy and practice implications. Addressing sociocultural and religious barriers to following medical advice provided for childbirth requires multilevel, community-driven strategies. Health education campaigns should be co-designed with relevant community stakeholders such as traditional leaders, religious leaders, influential community groups and women’s groups to ensure contextual relevance, cultural sensitivity and local acceptability. Embedding maternal health messaging into religious gatherings, mass media, and community gatherings may help to shift norms and improve engagement with maternal health advice and services. Government policies could prioritise a sustainable system for training and embedding women as peer counsellors in antenatal education programmes as evidence suggests that women are very receptive to advice from their peers [78]. Additionally, the government could also prioritise the inclusion of male partners and family decision-makers in planned interventions, as their influence is critical in shaping maternal health choices. Future research should evaluate the impact of culturally tailored interventions on maternal health behaviours. Key areas include the adaptation of health communication to cultural contexts, integrating faith-based messaging with medical advice, and conducting implementation research to evaluate their effectiveness in improving maternal and perinatal health outcomes.

### Strengths and limitations of the study

A key strength of this study lies in the diversity of the participants, representing a range of personal and professional backgrounds and experiences, enabling different perspectives to be combined (data triangulation) which helped to increase the validity of the findings [79]. UGA, AO, VY and JMc independently analysed the data, combining insider and outsider cultural perspectives to increase the credibility of findings (investigator triangulation). It was a limitation that although data saturation was achieved with the healthcare workers, recruitment of women had to stop before data saturation was reached, due to limited resources. We also excluded some areas in Imo state during participant recruitment due to the unstable political situation at the point of data collection. Participants shared both their first-hand experience of the discussion topic and their perceptions of what other women did. However, comparison between the first and second-hand reports showed that women admitted personally deviating from expected norms in pregnancy, like missing routine medications or infrequent attendance at antenatal care, which suggests that social desirability bias was likely to be minimal [80]. The research team engaged in reflexive practices to consider our assumptions and regularly discussed them to increase the credibility and trustworthiness of our analysis.

## Conclusions

This study provides insights into the sociocultural barriers to following medical advice during pregnancy in Nigeria, which include lack of trust in health professionals, power dynamics within a woman’s family, and entrenched cultural and religious beliefs that oppose medical intervention and especially caesarean birth. Women do not make decisions about pregnancy and birth in isolation, but are significantly influenced by their family, community and cultural norms. Culturally sensitive interventions deploying a multifaceted, whole-community approach are therefore needed to improve maternal and perinatal outcomes in Nigeria. These should aim to rebuild community trust in the health system, include family decision makers in antenatal care, and engage the whole community in wider maternal health education, including partnerships with religious and other local leaders. In settings like Imo State, which have high levels of access to maternity care, addressing these sociocultural barriers is crucial for translating maternal care access into improved maternal and newborn health outcomes, including reductions in stillbirths.

## Electronic supplementary material

Below is the link to the electronic supplementary material.

Supplementary Material 1

## Data Availability

The entire dataset for this study has not been included in the supplementary files as other papers are currently being developed from it. Hence, the data cannot be made freely available through data-sharing websites until all papers are published. Further inquiries can be directed to the corresponding author.

## Abbreviations

APH: Antepartum hemorrhage
CHW: Community health work
CS: Caesarean section
HHW: Hospital-based health worker
NHIS: National health insurance scheme
NM: Near-miss
