# Supplementary material for: “Is she pregnant with Jesus?” exploring sociocultural obstacles to following medical advice in the context of stillbirth prevention in Nigeria
Source: BMC Pregnancy Childbirth. 2025 May 20;25:593. doi: 10.1186/s12884-025-07646-5 (PMC12090445; doi:10.1186/s12884-025-07646-5)
Supplement: Supplementary file 1 — Supplementary Material 1 [file 12884_2025_7646_MOESM1_ESM.docx]

**TOPIC GUIDE FOR IN-DEPTH INTERVIEWS - Women**

**Introduction**

Pleasantries and Introduction.

Thank you for speaking with me today, I am sorry for your loss. I am meeting you today as I am currently studying pregnancies and their outcomes in Imo state. You may be aware that there are many pregnancies resulting in the death of mothers or babies in Imo state, and this affects many families some of which you may know personally. We are doing this research because we hope it may help healthcare providers understand how to improve support and outcomes for women in Imo State and will these be applicable elsewhere in Nigeria. .

My particular interest is the death of babies in the womb after 28 weeks of pregnancy. You have been invited to participate in this study because in the last two years, you have carried a pregnancy past 28 weeks. What we will discuss here will remain confidential.

I will start by asking you about the ease of receiving hospital care during your pregnancy, then we will look at the support you had through pregnancy especially when you had concerns for your health or your baby’s health. I will finish by asking your advice on what sort of support pregnant women in Imo state will need to improve health outcomes based on your experiences. The entire session should take about 45-60 minutes. With your permission, I would like to record this session solely for the purpose of note taking, after I analyse the results, I may include anonymised quotes from our conversation in my report and publication but nothing you say will be linked or traceable to you.

***Questions to be discussed***

*Questions will be modified slightly for women who had stillbirths in order to address the sensitivity related to the topic.*

**Women who had stillbirths**

The death of a baby in the womb is a very difficult experience for all women. And speaking about it is hard too. I am speaking with you today to understand and explore ways that healthcare providers might prevent the death of other babies in the future. Please know that you can ask me to stop or you can request for a break at anytime, if the conversation becomes too difficult for you.

*Exploring perceptions of healthcare access while pregnant*

1. Could you start by telling me a little bit about yourself and your family? (Probes: number/ages of children, employment, with a partner). About your baby that died,
2. Did you give your baby a name (and if yes then ask if it’s okay to use it) and if not, ask: how would you want me to refer to the baby throughout our conversation?
3. Thinking about your pregnancy for baby (name), how did you find and decide what hospital to use for your pregnancy and childbirth? Probe: Why did you decide to use this hospital? How did you find out what you needed to know to make this decision? Are there other factors some other women in your community may consider in choosing a hospital which may not be important for you?
4. How often did you visit the hospital for antenatal visits? Probe: what affected your decision about how often to go for antenatal visits? Are there other factors for some other women in your community? Does it make a difference which particular service or test is being offered at that antenatal appointment for women to keep an appointment or not?(e.g ultrasound scan, blood tests, urine tests etc). If there were interventions offering XX services (depending on the responses e.g. free services if the cost was mentioned as a barrier) do you think women will be open to using the services?

*Exploring the factors influencing healthcare and self-care decisions during pregnancy*

1. (Particularly if first baby) What did you already know about pregnancy and possible complications at that time? Probe: where or from who did you get this information?
2. Were there times during your pregnancy when you had questions or concerns about your health and that of your baby when you weren’t in the clinic on your ANC days? How did you handle the concerns/questions? **Probe:** was this person/resource always available for all such occasions?
3. Some women consider pregnancy to be a very stressful period of life. Looking back now, who supported you regarding your health and that of your baby outside of your clinic days? **Probe:** In what ways did they help you that made an impact on your health or your baby’s health? What other sort of support would you have wanted? Are there other ways health workers or other health workers could have supported your health and that of your baby during your pregnancy outside of clinic days?

*Exploring reflections about stillbirth and general recommendations*

1. At what point did you realise that something was not right during your pregnancy for baby (name)? Probe: What happened then? I realise that this is a very difficult experience, but looking back now, do you think any help you could have received while you were still at home or on your way to the hospital would have made any difference?
2. Based on your experience, what will you consider the most valuable advice you can give other women, health workers and the government to prevent future occurrences of stillbirth within your community? (If appropriate add probes to ask her opinion on what other women have said)
3. Is there anything else you would like to tell me about your experience?

**Women who had near-misses**

*Exploring perceptions of healthcare access while pregnant*

1. Could you start by telling me a little bit about yourself and your family? (Probes: number/ages of children, employment, with a partner). How would you want me to refer to your baby throughout our conversation?
2. Thinking about your pregnancy for baby (name) how easy was it for you to find and decide what hospital to use for your pregnancy and childbirth? Probe: What factors made you decide to use this hospital? Do you think other women consider the same factors? Are there other factors some other women in your community may consider in choosing a hospital which may not be a factor for you?
3. How often did you visit the hospital for antenatal visits? Probe: what affected your decision about how often to go for antenatal visits? Are there other factors for some other women in your community? Does it make a difference which particular service or test is being offered at that antenatal appointment for women to adhere to appointment or not?(e.g ultrasound scan, blood tests, urine tests etc). If there were interventions offering XX services (depending on the responses e.g. free services if the cost was mentioned as a barrier) do you think women will be open to using the services?

*Exploring the factors influencing healthcare and self-care decisions during pregnancy*

1. (Particularly if first baby) What did you already know about pregnancy and possible complications at that time? Probe: where or from who did you get this information?
2. Were there times during your pregnancy when you had questions or concerns about your health and that of your baby outside of your ANC days? How did you handle the concerns/questions? **Probe:** was this person/resource always available for all such occasions?
3. Some women consider pregnancy to be a very stressful period of life. Looking back now, who supported you regarding your health and that of your baby outside of your clinic days? **Probe:** In what ways did they help you that made an impact on your health or your baby’s health? What other sort of support would you have wanted? Are there other ways health workers or other health workers could have supported your health and that of your baby during your pregnancy outside of clinic days?

*Exploring reflections about the near-miss and general recommendations*

1. At what point did you realise that something was not right during your pregnancy for baby (name)? Probe: What happened then? After you realised you (your baby) were not doing okay, what were the key things that may have happened or the things you or someone else did before you arrived at the hospital that made a difference?
2. Based on your experience, what will you consider the most valuable advice you can give other women, health workers and the government to prevent future occurrences of stillbirth within your community?
3. Is there anything else you would like to tell me about your experience?

**TOPIC GUIDE FOR INDEPTH INTERVIEWS – Health workers**

**Introduction**

Pleasantries and Introduction.

I am meeting with you today as I am currently studying stillbirth occurrences in Imo State. It is unfortunate that Nigeria still has an unacceptably high burden of stillbirths with far-reaching impact on families and the health system. You have been invited to participate in this study because you are a health worker, and you work closely with women through the course of pregnancy and birth. What we will discuss here will remain confidential.

I will start by asking your opinion about community factors leading to stillbirths, perceived impact of stillbirth occurrence on health users and finally your perspectives on community-based strategies that can help reduce stillbirth occurrence. The entire session should take about 45 - 60 minutes. With your permission, I would like to record this session solely for the purpose of note taking, nothing you say on record will be linked to you.

***Questions to be discussed***

*Exploring reflections on community influence on stillbirths*

1. Can you tell me about your professional background and your current role? Probe: How long have you been in this role? How often do you work with women who experience stillbirths or near-misses? Have you worked in other places outside of Imo state?
2. To what extent do you think that factors outside of the hospital settings influence the occurrence of stillbirths in this area ? **Probe**: Why do you think these factors affect women in this area? Can you explain a bit more? Has this changed over the time you have worked here? Is this different in any other areas you have worked in?
3. For the women that access maternal health services in your hospital (or in hospitals in this locality – for CHWs), what are the factors or persons outside of the health workers that influence or shape their health habits? **Probe**: does this influence affect their outcomes? Can these influences be controlled (where negative) or improved (where positive) through any actions of the health workers or any of our health agencies and regulators? Probe: (give examples of factors if necessary)

*Exploring reflections about community impact following stillbirth and roles in prevention*

I am aware you are a health worker, but you also live within communities and over the years, you may have developed your opinions about the next few questions.

1. When a woman has had a stillbirth in a health facility, how do you think the news affects the people within her social circles in the community? **Probe:** Do you think it can affect future health-seeking habits of people who may hear the story? (If negative impact) Is there anything that can be done at the health system or community level to minimise the impact?
2. What role do women, their family and communities can have in preventing stillbirths? ***Probe:*** Do you think women and their families know they can contribute in the way you have described? Why is this so? What are the enablers and barriers they may face to contributing?
3. What role do communities and community organisations (including faith groups) have in preventing stillbirths? ***Probe:*** Are they currently playing these roles in the way you have described? What are the enablers and barriers they may face to contributing? Who else has a role?

*Reflections on facility and community prevention collaborations*

1. What are your thoughts on how the efforts to reduce stillbirth at the health facilities can be complimented by interventions at the community level? **Probe**: Are there example of such interventions you have seen work elsewhere? How can these interventions be nurtured and sustained? Probe: (give examples of interventions if necessary)
2. Is there anything else you want to add about preventing stillbirths in your local area?
